# Stimulation of MMP-9 of oral epithelial cells by areca nut extract is related to TGF-β/Smad2-dependent and –independent pathways and prevented by betel leaf extract, hydroxychavicol and melatonin

**DOI:** 10.18632/aging.102565

**Published:** 2019-12-12

**Authors:** Mei-Chi Chang, Yu-Hwa Pan, Hsyueh-Liang Wu, Yi-Jie Lu, Wan-Chuen Liao, Chien-Yang Yeh, Jang-Jaer Lee, Jiiang-Huei Jeng

**Affiliations:** 1 Chang-Gung University of Science and Technology, Kwei-Shan, Taoyuan, Taiwan; 2 Department of Dentistry, Chang Gung Memorial Hospital, Taipei, Taiwan; 3 Department of Chemistry, National Taiwan Normal University, Taipei, Taiwan; 4 Graduate Institute of Oral Biology, National Taiwan University Medical College, Taipei, Taiwan; 5 School of Dentistry, National Taiwan University Medical College, and Department of Dentistry, National Taiwan University Hospital, Taipei, Taiwan

**Keywords:** areca nut, betel leaf, hydroxychavicol, MMP-9, oral cancer

## Abstract

Background: There are 200-600 million betel quid (BQ) chewers in the world. BQ increases oral cancer risk. Matrix metalloproteinase-9 (MMP-9) is responsible for matrix degradation, cancer invasion and metastasis. Whether areca nut extract (ANE), a BQ component, stimulates MMP-9 secretion, and the related signaling pathways awaits investigation.

Results: ANE (but not arecoline) stimulated MMP-9 production of gingival keratinocytes and SAS cancer epithelial cells. ANE stimulated TGF-β1, p-Smad2, and p-TAK1 protein expression. ANE-induced MMP-9 production/expression in SAS cells can be attenuated by SB431542 (ALK5/Smad2 inhibitor), 5Z-7-Oxozeaenol (TAK1 inhibitor), catalase, PD153035 (EGFR tyrosine kinase inhibitor), AG490 (JAK inhibitor), U0126 (MEK/ERK inhibitor), LY294002 (PI3K/Akt inhibitor), betel leaf (PBL) extract, and hydroxychavicol (HC, a PBL component), and melatonin, but not by aspirin.

Conclusions: AN components contribute to oral carcinogenesis by stimulating MMP-9 secretion, thus enhancing tumor invasion/metastasis. These events are related to reactive oxygen species, TGF-β1, Smad2-dependent and –independent signaling, but not COX. These signaling molecules can be biomarkers of BQ carcinogenesis. PBL, HC and melatonin and other targeting therapy can be used for oral cancer treatment.

Methods: ANE-induced MMP-9 expression/secretion of oral epithelial cells and related TGF-β1, Smad-dependent and –independent signaling were studied by MTT assay, RT-PCR, western blotting, immunofluorescent staining, and ELISA.

## INTRODUCTION

Betel quid (BQ) chewing is popular in Taiwan and many other countries. There are about 2.8 million BQ chewers in Taiwan and 200-600 million BQ chewers in the world [1, 2]. In Taiwan, BQ contains mainly areca nut (AN), lime, *piper betle* inflorescence with/without betel leaf (*piper betle* leaf). The major chemical components of AN is alkaloids (arecoline, arecaidine, guvacoline, guvacine etc.), catechol, catechin, polyphenols (flavonol, tannin), minerals (Cu, Fe etc.), carbohydrate, fat, protein, crude fibers etc. [1, 2]. ANE, arecoline, reactive oxygen species generated during oxidation of ANE, and the AN-derived nitrosamines are considered to be the possibly carcinogens. They exhibit genotoxicity, mutagenicity and cell transformation capacities in different assay systems [1, 2]. Clinically, BQ chewing increases the risk of oral leukoplakia, oral lichenoid lesions, oral submucous fibrosis (OSF) and oral squamous cell carcinoma (OSCC) [1, 2]. BQ ingredients are involved in the initiation and promotion of oral cancer by induction of DNA damage, chromosomal aberration, tissue inflammation, fibrosis and malignant transformation [1, 3]. However, limited information is known about the BQ components in tumor invasion, metastasis and progression.

Matrix metalloproteinases (MMPs) play important roles in tissue inflammation, tumor invasion and metastasis, by degradation of extracellular matrix [4, 5]. OSCC expresses higher level of MMP-2 and MMP-9 [6]. It is intriguing to know whether BQ components may affect MMPs expression/production and contribute to oral carcinogenesis. Recently, areca nut extract (ANE) activates MMP-9, but not MMP-2 expression in gingival epithelial cells, that can be inhibited by NF-kB inhibitor and curcumin [7]. ANE also stimulates MMP-9, but decreases tissue inhibitor metalloproteinase-1 (TIMP-1) and TIMP-2 secretion of SAS tongue cancer epithelial cells [8]. Salivary MMP-9 levels and MMP-2 and MMP-9 mRNA expression in OSCC are markedly increased and related to lymph node metastasis [9]. All the above reveal the importance of MMPs in oral carcinogenesis.

Previously we have found that AN components stimulates cytochrome P450, reactive oxygen species (ROS), check point kinase-1/2 (Chk1/Chk2), a disintegrin and metalloproteinases (ADAMs), epidermal growth factor/epidermal growth factor receptor (EGF/EGFR), Ras, Src, Janus kinase (JAK), mitogen-activated protein kinase kinase (MEK)/extracellular signal-regulated kinase (ERK), phosphoinositide 3-kinase (PI3K)/protein kinase B (Akt) signaling, cell cycle arrest, apoptosis and release of various inflammatory mediators such as 8-isoprostane, interleukin-1α (IL-1α), prostaglandin E_2_ (PGE_2_), IL-6, IL-8, etc. in different kind of cells [3, 10–14]. BQ components, ANE, and arecoline, are able to stimulate TGF-β signaling, and both OSCC and OSF tissues expressed higher level of TGF-β [15, 16]. ROS, TGF-β, tumor necrosis factor-α (TNF-α), IL-1α and IL-1β have been shown to induce Smad-dependent (ALK5/Smad) and -independent (transforming growth factor β-activated kinase-1, TAK1) signaling [17, 18]. TAK1 further induces downstream signaling pathways such as ROS, EGFR, mitogen-activated protein kinases (MAPKs), Akt, and nuclear factor kappa-B (NF-κB) etc. to regulate a number of cellular and clinical events, e.g., tissue inflammation/inflammatory diseases, cell death/tissue homeostasis, rheumatoid arthritis and carcinogenesis/cancer etc. [18–20].

To know whether BQ chewing and AN components can promote cancer progression, invasion and metastasis, it is interesting to know whether AN components may stimulate MMP-9 expression in oral epithelial cells and the role of TGF-β1/Smad2-dependent and Smad-independent (TAK1 and other related signal transduction) pathways. Moreover, one clinically critical question is whether including of PBL into BQ may enhance or decrease its carcinogenicity that is important for development of health policy and disease prevention for the country. PBL contains chemicals mainly hydroxychavicol (HC), eugenol, chavicol and carotene [2], and are shown to exhibit potential anti-mutagenic and anti-carcinogenic effect [2]. PBL extract and HC are found to have anti-oxidant, anti-inflammatory and anti-platelet effect possibly via scavenging ROS and inhibition of cyclooxygenase (COX) [21, 22]. Moreover, melatonin has been shown to have anti-cancer effects by mitigating the initiation, progression and metastasis of cancer development and growth possibly via receptor-dependent and –independent manners [23, 24]. Melatonin is shown to scavenge reactive oxygen species (ROS)/redox regulation, inhibition of signaling molecules, increase sensitivity of cancer cells to chemotherapeutic drugs, modulating of non-coding RNA, control of angiogenesis and apoptosis etc. [23, 24]. Since melatonin is present as natural hormone in human body and used clinically for insomnia/sleep improvement in different conditions [24], its preventive effect on ANE-induced toxicity is useful.

Since ROS and COX are associated with ANE toxicity and tissue inflammation is consistently found in tissues of OSF and OSCC [1–3, 10, 11], clarifying the preventive efficacy of betel leaf (*piper betle* leaf [PBL], hydroxychavicol (HC), aspirin and melatonin, on the ANE-induced alterations in oral mucosal cells is valuable for cancer prevention and treatment. Results of this study can be helpful for targeting therapy of OSCC in the future.

## RESULTS

### Effect of ANE and arecoline on MMP-9 mRNA expression of SAS cells

**Figure 1:**
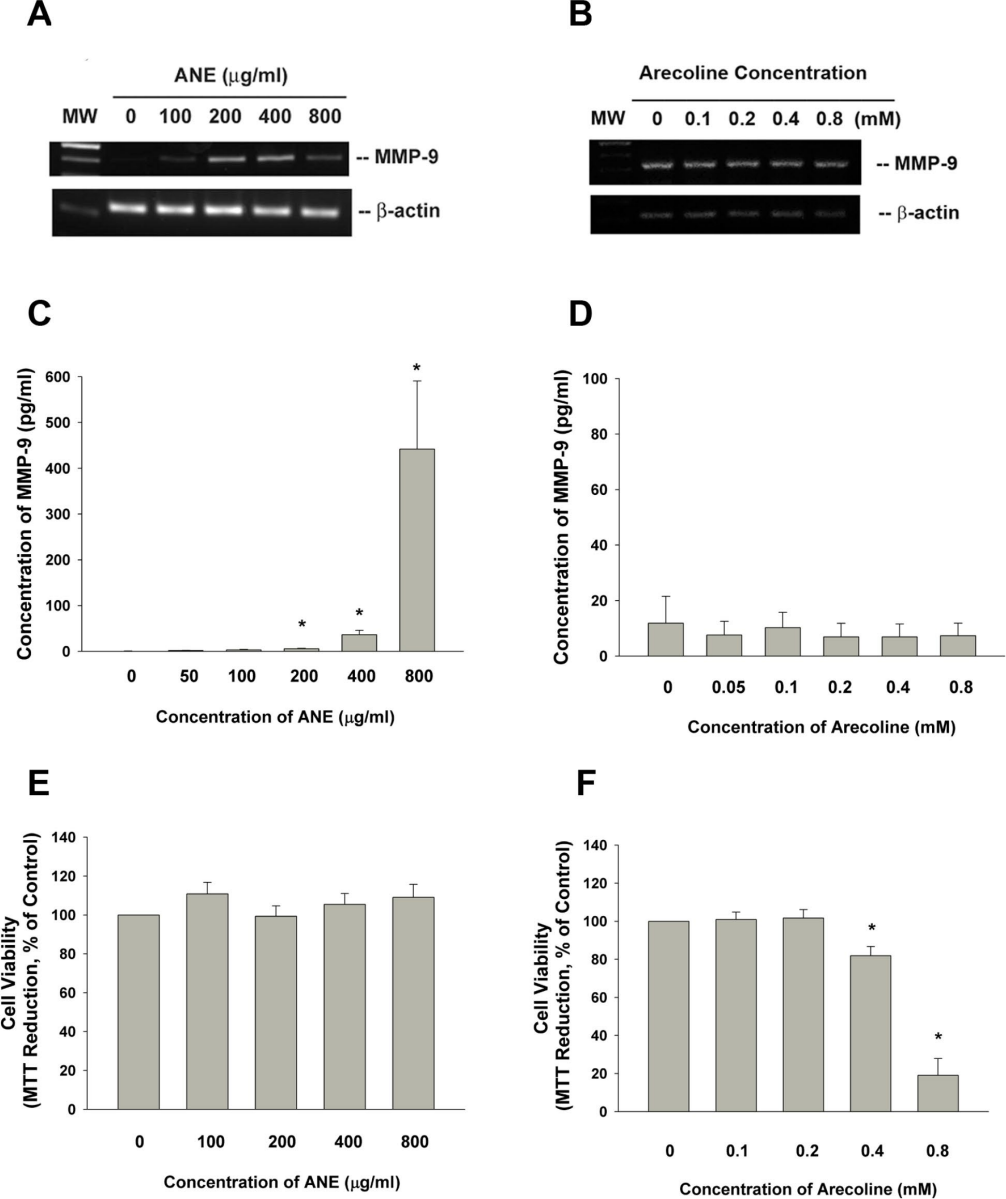
**Effect of ANE and arecoline on MMP-9 expression/secretion of SAS cells.** (**A**) Effect of ANE (100-800 μg/ml) and (**B**) Arecoline (0.1-0.8 mM) on MMP-9 mRNA expression in SAS oral cancer epithelial cells as analyzed by RT-PCR. One representative semi-quantitative RT-PCR result was shown. (**C**) Effect of ANE (50-800 μg/ml) on MMP-9 secretion of SAS cells, after 3 days of exposure, (**D**) Effect of arecoline (0.05-0.8 mM) on MMP-9 secretion of SAS cells after 3 days of exposure, Results were expressed as Mean ± SE (pg/ml), (**E**) Effect of ANE (100-800 μg/ml) on cytotoxicity to SAS cells, (**F**) Effect of arecoline (0.1-0.8 mM) on cytotoxicity to SAS cells, Results were expressed as percentage of control (as 100%, Mean ± SE). *denotes statistically significant difference between groups.

We recently found the stimulation of MMP-9 production of oral epithelial cells by ANE at non-cytotoxic concentrations as analyzed by enzyme-linked immunosorbent assay (ELISA). On the other hand, arecoline showed no marked stimulatory effect on MMP-9 production of oral epithelial cells, even decrease MMP-9 production possibly due to cytotoxicity at concentrations of 0.4 and 0.8 mM [8]. Accordingly, ANE also stimulated MMP-9 secretion of gingival keratinocytes (GK) (data not shown). ANE also induced MMP-9 mRNA expression of oral epithelial cells as analyzed by reverse transcription-polymerase chain reaction (RT-PCR) (Figure 1A). But arecoline showed no marked stimulatory effect on MMP-9 expression (Figure 1B). Accordingly, ANE stimulated the MMP-9 secretion of SAS cells after 3 days of exposure (Figure 1C), whereas arecoline showed slight decrease of MMP-9 secretion (Figure 1D). At tested concentrations, ANE showed no evident changes on viability of SAS cells (Figure 1E). Arecoline suppressed the viability of SAS cells at concentrations higher than 0.4 mM (Figure 1F).

### Role of TGF-β1 and Smad2 signaling in ANE-induced MMP-9 secretion of GK and SAS cells

**Figure 2:**
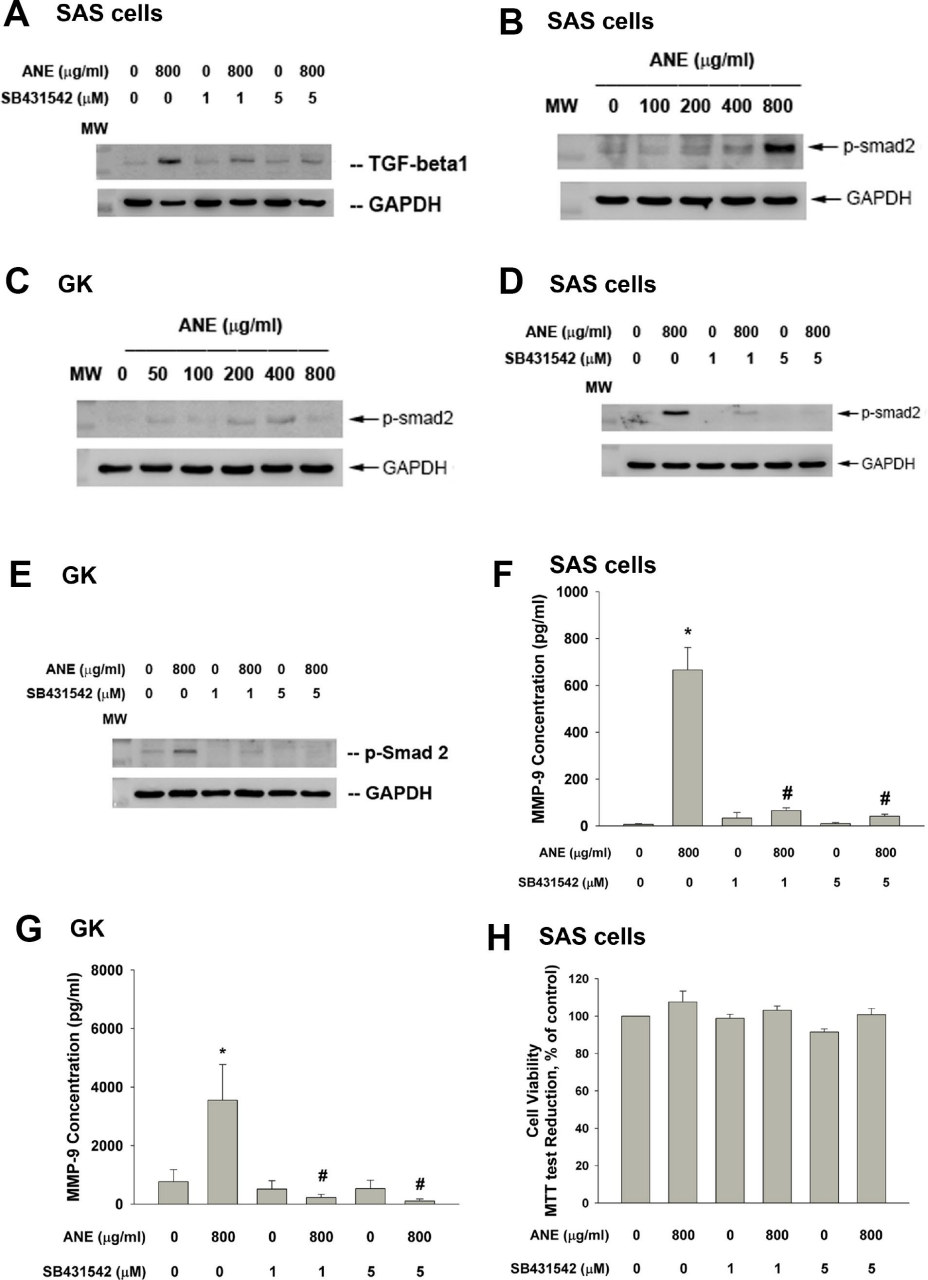
**Role of TGF-β and Smad2 signaling on ANE-induced MMP-9 expression/secretion of oral epithelial cells.** (**A**) Stimulation of TGF-β protein expression of SAS cells by ANE (800 μg/ml) and its attenuation by SB431542 (1 and 5 μM), (**B**) ANE (100-800 μg/ml) stimulated Smad2 phosphorylation of SAS cells after 24-hr of exposure, (**C**) ANE (50-800 μg/ml) stimulated Smad2 phosphorylation of GK after 24-hr of exposure, (**D**) SB431542 (1 and 5 μM) attenuated the ANE (800 μg/ml)-induced p-Smad2 expression of SAS cells, (**E**) SB431542 attenuated the ANE (800 μg/ml)-induced p-Smad2 expression of GK, (**F**) SB431542 prevented the ANE (800 μg/ml)-induced MMP-9 secretion of SAS cells, (**G**) SB431542 prevented the ANE (800 μg/ml)-induced MMP-9 secretion of GK, (**H**) SB431542 showed little effect on ANE (800 μg/ml)-induced cytotoxicity of SAS cells (as % of control, 100%). *denotes statistically significant difference when compared with control. #denotes statistically significant difference when compared with ANE-treated group.

ANE stimulated TGF-β1 protein expression and this event can be inhibited by SB431542, an ALK5/Smad2/3 signaling inhibitor (Figure 2A). ANE induced the Smad2 phosphorylation in both SAS cells and primary GK (Figure 2B, 2C). SB431542 markedly attenuated the ANE-induced Smad2 phosphorylation in SAS cells and GK (Figure 2D, 2E). Intriguingly, SB431542 also prevented the ANE-induced MMP-9 secretion of both SAS cells and GK (Figure 2F, 2G). In this experiment condition, SB431542 showed little effect on the viability of SAS cells (Figure 2H).

### Role of TAK1 in ANE-induced MMP-9 expression/secretion

**Figure 3:**
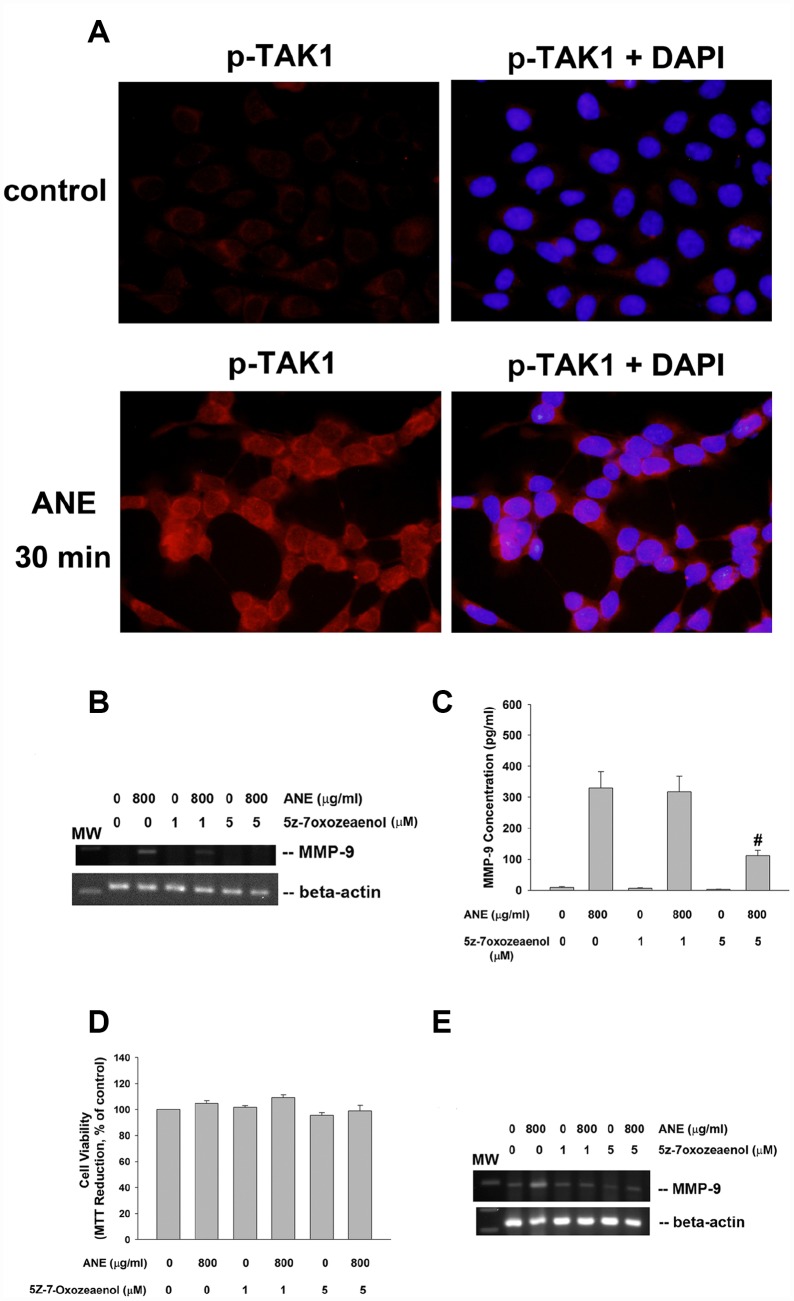
**Role of TAK1 activation in ANE-induced MMP-9 expression/secretion.** (**A**) Stimulation of p-TAK1 activation of SAS cells by ANE. (upper) solvent control, and (lower) SAS cells treated by ANE (800 μg/ml) for 30 min. Pictures of p-TAK1 fluorescence (left) & merged with DAPI (right). Effects of 5Z-7-Oxozeaenol (1 and 5 μM) on (**B**) ANE (800 μg/ml)-induced MMP-9 mRNA expression of SAS cells as analyzed by semi-quantitative RT-PCR. One representative result was shown, and (**C**) ANE (800 μg/ml)-induced MMP-9 secretion in SAS cells as analyzed by ELISA. (**D**) Effect of ANE with/without 5Z-7-Oxozeaenol on the viability of SAS cells (as % of control, 100%), (**E**) ANE-induced MMP-9 mRNA expression of GK as analyzed by semi-quantitative RT-PCR.

Interestingly we found the activation of TAK1 by ANE. ANE rapidly stimulated p-TAK1 protein expression of SAS cells within 30 min of exposure, as shown by increase in red fluorescence (Figure 3A). To know further about the role of TAK1 in ANE-induced MMP-9 expression and secretion of oral mucosal cell, we intriguingly found that 5Z-7-Oxozeaenol (a TAK1 inhibitor) prevented the ANE-induced MMP-9 mRNA expression of SAS cells (Figure 3B). Accordingly, 5z-7oxozeaenol also effectively attenuated the ANE-induced MMP-9 secretion of SAS cells (Figure 3C). At this experimental condition, 5Z-7-Oxozeaenol showed no marked changes on the viability of SAS cells (Figure 3D). Similarly, 5z-7oxozeaenol also prevented the ANE-induced MMP-9 mRNA expression (Figure 3E) and secretion of GK (data not shown). 5Z-7-Oxozeaenol also prevented the ANE-induced Smad2 phosphorylation in SAS cells (data not shown), suggesting the presence of crosstalk between different signaling pathways.

### Effect of Catalase, PD153035 and AG490 on ANE-induced MMP-9 production

**Figure 4:**
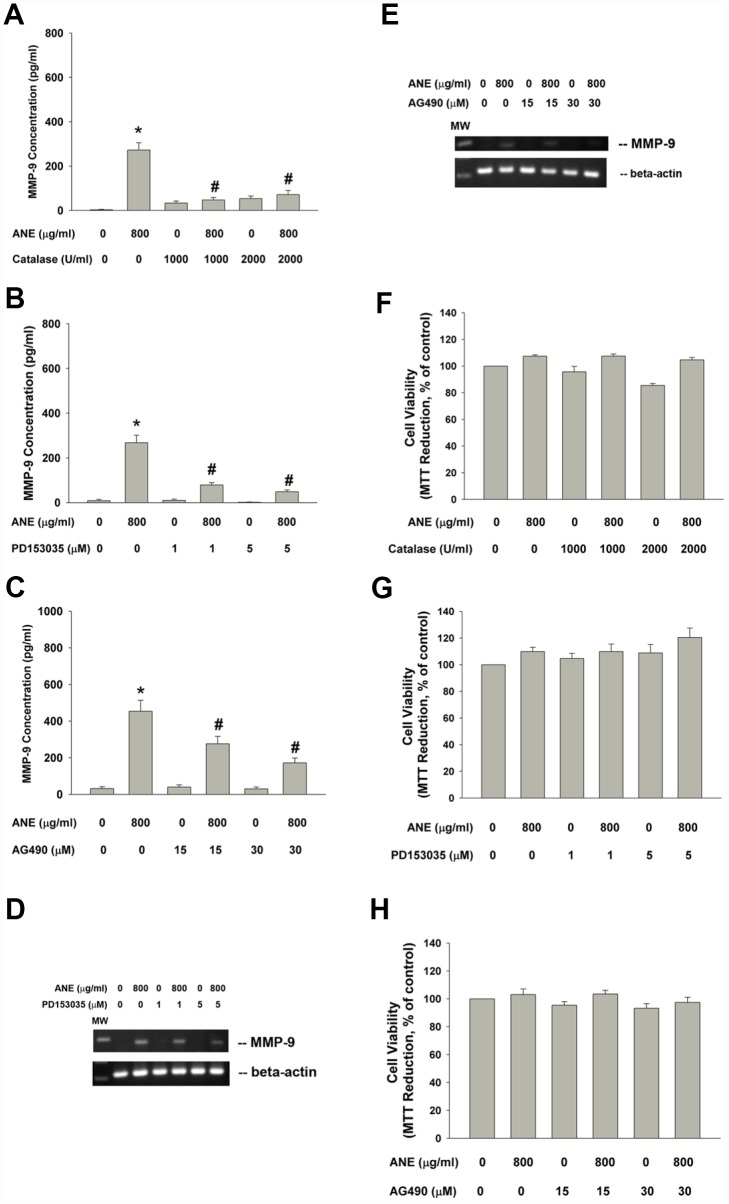
**Effect of catalase, PD153035 and AG490 on ANE-induced MMP-9 expression/secretion of SAS cells. Effect of** (**A**) catalase (1000 and 2000 U/ml), (**B**) PD153035 (1 and 5 μM) and (**C**) AG490 (15 and 30 μM) on ANE (800 μg/ml)-induced MMP-9 secretion in SAS oral cancer epithelial cells. Results were expressed as Mean ± SE (pg/ml). (**D**) Effect of PD153035 and (**E**) Effect of AG490 on ANE (800 μg/ml)-induced MMP-9 mRNA expression, Effect of (**F**) catalase, (**G**) PD153035 and (**H**) AG490 on ANE (800 μg/ml)-induced changes of cell viability of SAS oral cancer epithelial cells. (Mean ± SE, % of control). *denotes statistically significant difference when compared with control. #denotes statistically significant difference when compared with ANE-treated group.

ANE has been shown to stimulate ROS production and glutathione (GSH) depletion [10], we therefore tested and found that catalase attenuated the ANE-induced MMP-9 production of oral epithelial cells (Figure 4A). Since ROS has been shown to be important signaling molecules, we further tested the possible EGFR and JAK signaling pathways in this event. Intriguingly, we found that PD153035 (a EGFR antagonist) evidently attenuated the ANE-induced MMP-9 production of SAS cells (Figure 4B). In addition, AG490 (a JAK inhibitor) was also effective to inhibit the ANE-induced MMP-9 production of SAS cells (Figure 4C). Accordingly, PD153035 and AG490 also attenuated the ANE-induced MMP-9 mRNA expression of SAS cells (Figure 4D, 4E). At these experimental conditions, catalase, PS153035 and AG490 showed no marked effect on the viability of SAS cells (Figure 4F, 4G, 4H), as analyzed by 3-(4,5-dimethylthiazol-2-yl)-2,5- diphenyltetrazolium bromide (MTT) assay (a mitochondrial enzyme activity marker).

### Role of PI3K/Akt and MEK/ERK in ANE-induced MMP-9 expression/secretion

**Figure 5:**
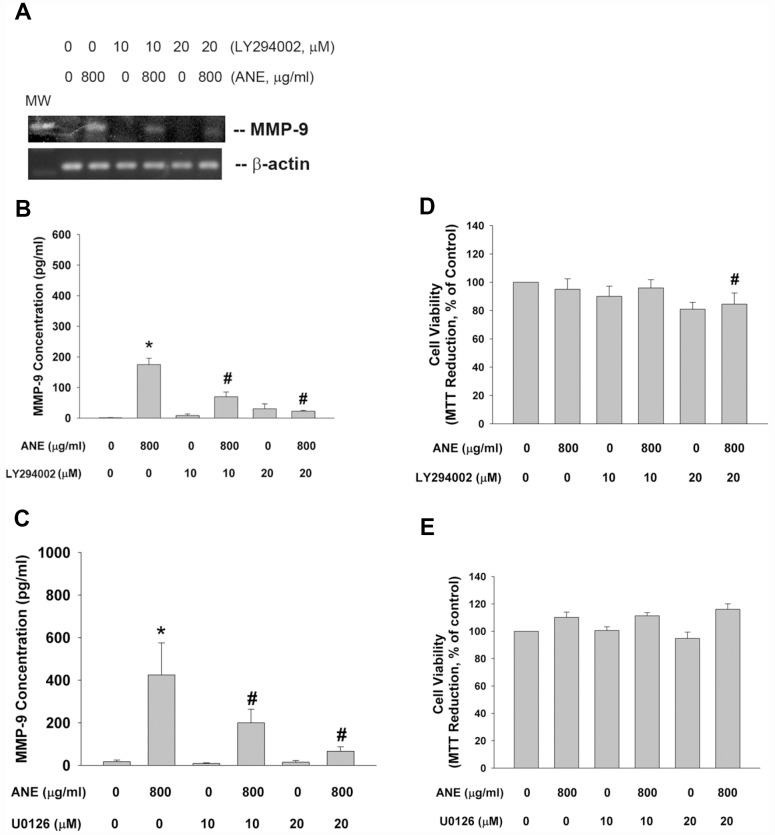
**Effect of LY294002, and U0126 on ANE-induced MMP-9 expression/secretion.** (**A**) Effect of LY294002 (10 and 20 μM) on ANE (800 μg/ml)-induced MMP-9 mRNA expression as analyzed by semi-quantitative RT-PCR. One representative result was shown, (**B**) Effect of LY294002 on ANE (800 μg/ml)-induced MMP-9 secretion in SAS cells as analyzed by ELISA. (**C**) Effect of U0126 (10 and 20 μM) on ANE (800 μg/ml)-induced MMP-9 secretion in SAS cells as analyzed by ELISA. *denotes statistically significant difference when compared with control. #denotes statistically significant difference when compared with ANE-treated group.

PI3K/Akt and MEK-ERK pathways are main downstream signaling molecules of ROS, EGFR and JAK. We interestingly found that LY294002 (a PI3K/Akt inhibitor) was able to prevent the ANE-induced MMP-9 mRNA expression (Figure 5A). Accordingly, LY294002 also suppressed the ANE-induced MMP-9 production of SAS cells (Figure 5B). Similarly, U0126 (a MEK/ERK inhibitor) inhibited the ANE-induced MMP-9 production of SAS cells (Figure 5C). LY294002 and U0126 showed little effect on ANE-induced cytotoxicity (Figure 5D, 5E).

### Effect of betel leaf, hydroxychavicol and aspirin on ANE-induced MMP-9 production

**Figure 6:**
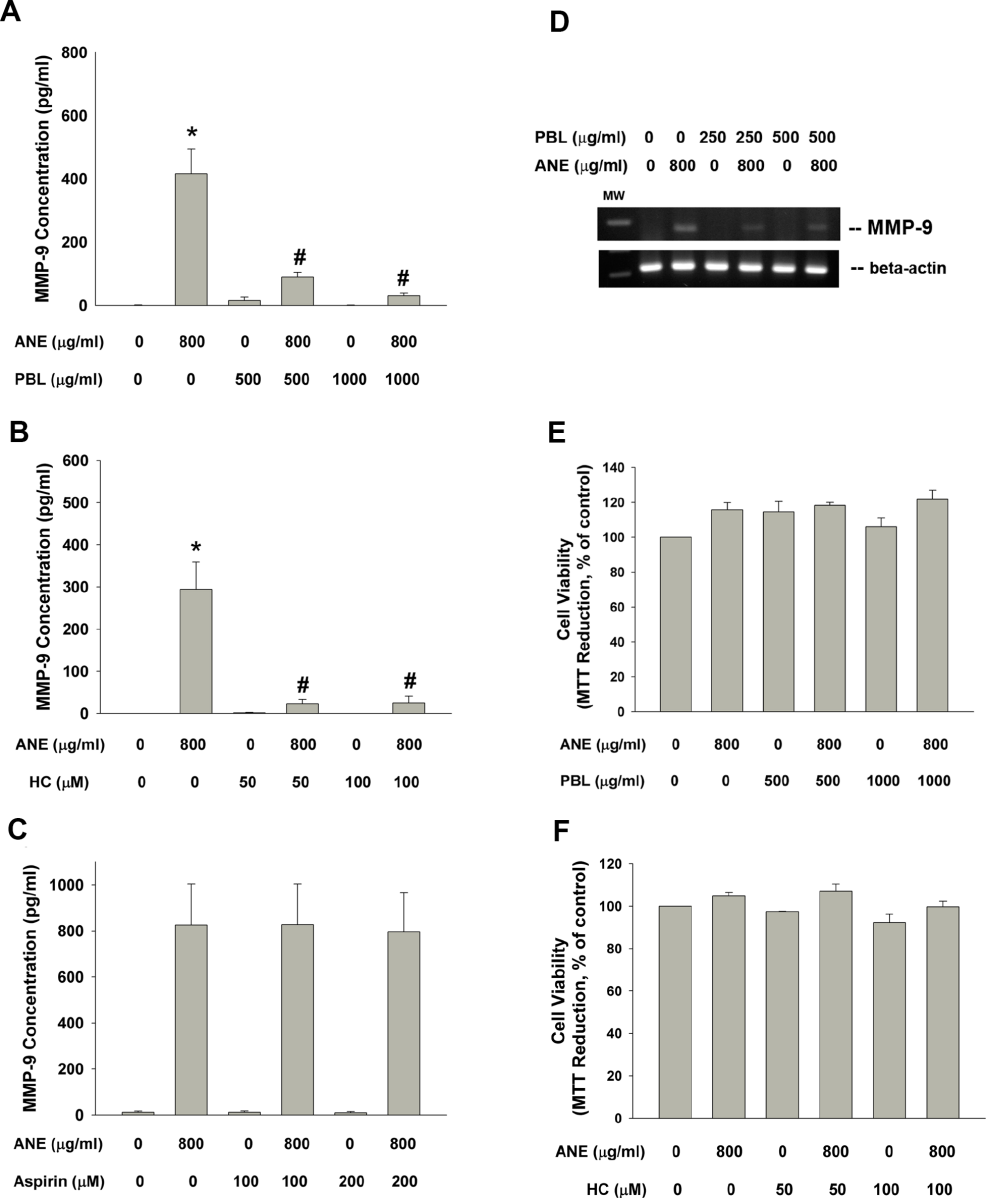
**Effect of PBL, HC and aspirin on ANE-induced MMP-9 secretion.** Effect of (**A**) PBL (500 and 1000 μg/ml), (**B**) HC (50 and 100 μM) and (**C**) aspirin (100 and 200 μM) on ANE (800 μg/ml)-induced MMP-9 secretion in SAS oral cancer epithelial cells. Results were expressed as Mean ± SE (pg/ml). (**D**) Effect of PBL on ANE-induced MMP-9 expression, and effect of (**E**) PBL and (**F**) HC on ANE-induced changes of cell viability of SAS cells (as % of control, Mean ± SE).

Betel leaf (*piper betle leaf*, PBL) extract and hydroxychavicol (HC) has been shown to exhibit antiplatelet, antioxidant and even anti-inflammatory properties and inhibit cyclooxygenase (COX) enzyme activities [21, 22]. Three PBL components, hydroxychavicol (HC), catechol and catechin showed no marked stimulatory effect on MMP-9 production of oral epithelial cells (data not shown). Interestingly PBL extract and HC effectively inhibited the ANE-induced MMP-9 production of SAS epithelial cells (Figure 6A, 6B). But aspirin (a cyclooxygenase [COX] inhibitor) showed little effect on ANE-induced MMP-9 production of SAS cells (Figure 6C). Accordingly, PBL extract attenuated the ANE-induced MMP-9 mRNA expression of SAS cells (Figure 6D). In this experimental conditions, PBL and HC showed little cytotoxicity to SAS cells (Figure 6E, 6F)

### Effect of melatonin on ANE-induced MMP-9 mRNA expression and protein secretion

**Figure 7:**
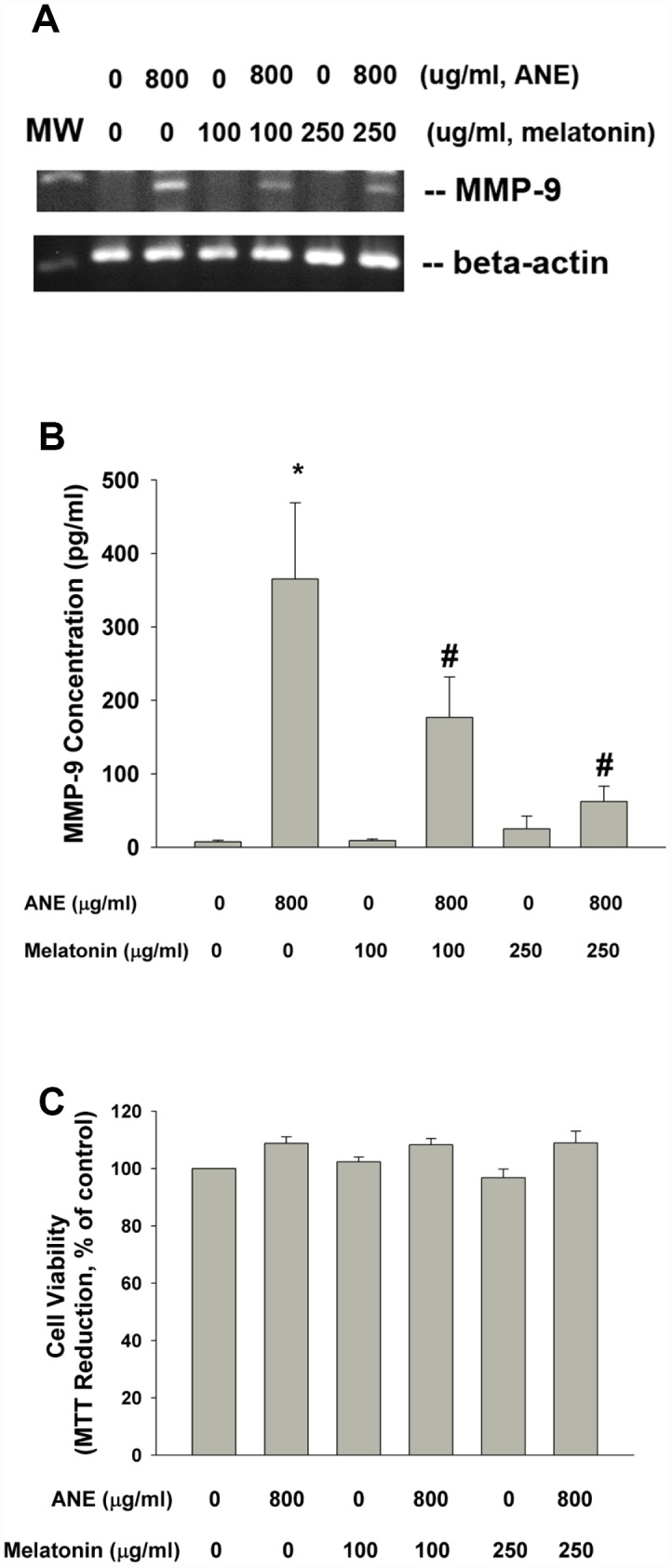
**Effect of melatonin on ANE-induced MMP-9 expression and secretion.** (**A**) Effect of melatonin (100 and 250 μg/ml) on ANE (800 μg/ml)-induced MMP-9 mRNA expression (one representative result was shown) and (**B**) Effect of melatonin on ANE-induced MMP-9 secretion in SAS oral cancer epithelial cells. Results were expressed as Mean ± SE (pg/ml). (**C**) Effect of melatonin on ANE-induced changes in cell viability of SAS cells (as % of control). *denotes statistically significant difference when compared with control. #denotes statistically significant difference when compared with ANE-treated group.

Pretreatment and co-incubation by melatonin (100 and 250 μg/ml) effectively prevented the ANE-induced MMP-9 mRNA expression and MMP-9 secretion into culture medium (Figure 7A, 7B). Melatonin showed little effect on ANE-induced changes of cell viability of SAS cells (Figure 7C).

## DISCUSSION

OSCC has been ranked as top 5 prevalent cancer in male of Taiwan. This can be due to oral habits such as BQ chewing, smoking and alcohol consumption in oral cancer patients. The 5-year survival rate of single primary OSCC patients is about 68% in Taiwan, a BQ chewing endemic area [25]. MMPs are involved in tumor invasion and metastasis and thus the prognosis/outcome of OSCC patients. Increased MMP-9 mRNA and protein expression are also reported in OSCC tissues [6, 9]. All these results suggest the contributory role of MMP-9 in oral carcinogenesis, tumor invasion and metastasis. Extracellular matrix homeostasis is important for wound healing, modulating keratinocyte behaviors, promoting keratinocyte migration, granulation tissue remodeling, and cancer [26]. Extracellular matrix turnover is tightly controlled by matrix metalloproteinases (MMP-2, MMP-9, collagenases etc.) and tissue inhibitors of metalloproteinases (TIMPs). TIMP-1 and TIMP-2 may inhibit the enzyme activity of MMP-2 and MMP-9 [27]. Degradation of basement membrane and interstitial connective tissues that contains mainly fibrillar collagens, and other ECM proteins such as proteoglycans, elastin, laminin, fibronectin etc. by enzymatic proteases around the tumor cells is an essential step for cancer invasion and metastasis [27, 28]. Over-expression of MMPs is associated with tumor growth, relapse, metastasis and overall survival in breast and liver cancers [27]. Since BQ chewing is one major risk factor of chewer’s mucosa, leukoedema, oral leukoplakia, ulcer and oral carcinogenesis [1, 29], it is interesting to know whether BQ components may stimulate MMP-9 expression and secretion in oral mucosal cells. In addition to stimulation of MMP-9 secretion in SAS cancer epithelial cells [8], ANE, but not arecoline, also stimulates MMP-9 mRNA expression of SAS cells and MMP-9 secretion of primary oral keratinocytes. Stimulation of MMP-9 production and expression by ANE indicates that BQ chewing possibly may contribute to the pathogenesis of chewer’s mucosa, leukoedema, ulcer, oral carcinogenesis and cancer progression by induction of MMP-9 and thus promote tumor invasion and metastasis. Since arecoline does not stimulate MMP-9 production, presence of other components in ANE that may stimulate MMP-9 production should be further clarified.

Recently AN components are reported to stimulate TGF-β, IL-1α and IL-1β [12, 13, 15, 16], that may potentially induce downstream transforming growth factor β-activated kinase-1 (TAK1) signaling. Interestingly we found that ANE increased TGF-β1 protein expression as well as the downstream p-Smad2 and p-TAK1 protein expression and signaling. SB431542 (an ALK5/Smad2/3 signaling inhibitor) attenuated the ANE-induced p-Smad2 and TGF-β1 protein expression as well as MMP-9 secretion. Moreover, 5Z-7-Oxozeaenol (a selective TAK1 inhibitor) prevented the ANE-induced MMP-9 mRNA expression and protein secretion of SAS and GK. These results suggest the involvement of both Smad-dependent and TAK1 signaling in ANE-induced MMP-9 activity of oral mucosal cells. Similarly, Yang et al. (2017) found the regulation of MMP-9 by TAK1 in gastric cancer cells [30]. Targeting and knockdown of TAK1 inhibits chemokine receptor 7, MMP-9, IL-8, COX-2, tumor growth and metastasis of breast cancer [31]. Similarly, TGF- β1 is shown to stimulate MMPs in hepatocellular carcinoma cells via ALK5/Smad and PI3K/Akt signaling [32, 33]. These results raise the possible targeting TAK1 and ALK5/Smad molecules in future prevention and treatment of oral cancer.

Reactive oxygen species (ROS) production shows reciprocal interaction with TAK1 activation. Whereas ROS activate TAK1/AMPK signaling, TAK1 activation also stimulates further ROS production in different kind of cells [17, 34]. Interactions and crosstalk between TGF-β/TAK1 and EGFR and JAK signaling are reported [19, 35]. Interestingly we also found the cross-talk between TAK1 and Smad2 signaling in ANE-induced events in oral epithelial cells (data not shown). BQ components may stimulate ROS production of oral mucosal cells [10, 13, 22]. Antioxidants such as N-acetylcysteine (NAC) and catalase are able to prevent the ANE- and arecoline-induced toxic events such as COX-2 expression, PGE2, IL-1α, cytotoxicity etc. [13, 22] as well as MMP-9 secretion of SAS cancer epithelial cells in this study. ANE can activate EGFR [12], and inhibition of EGFR and JAK signaling by PD153035 and AG490 also suppressed the ANE-induced MMP-9 production of SAS cancer cells. Similarly, ROS may stimulate COX-2 expression of synovial fibroblasts via TAK1 signaling [17]. Moreover, inhibition of EGFR and JAK may potentially prevent the ANE-induced ADAM17, IL-1α, PGE2 production, COX-2 expression, ADAM9 maturation, and the ANE-induced decline in keratin 5 and 14 [12, 13]. EGFR overexpression in head/neck SCC is reported and associated with tumor stages and tumor-free survival [36]. Further, alteration of JAK2/Stat3 related genes’ expression in late stage OSCC is also noted [37]. These indicate that ROS scavengers, EGFR and JAK targeting molecules can be potentially used for future therapy of OSCC.

Elevated expression of p-Akt in OSCC and possible induction by nicotine is reported [38]. BQ, ANE and arecoline may stimulate MEK/ERK and/or PI3K/Akt signaling, to induce heat shock protein (hsp) 47, IL-α, ADAM17, 8-isoprostane, and COX-2, but down-regulate involucrin expression of oral mucosal cells [11–14, 39]. Similarly, PI3K/Akt and MEK/ERK signaling pathways are also important for ANE-induced MMP-9 gene expression/production in oral epithelial cells. While arecoline stimulates PI3K/Akt to mediate hsp47 expression [39, 40], it does not stimulate MMP-9 expression/secretion of SAS epithelial cells [8]. While both ANE and arecoline may stimulate PI3K/akt signaling, their downstream reactive molecules are perhaps different and should be further addressed.

PBL is a popularly-used additive in BQ. Whether addition of PBL into BQ is beneficial or harmful to oral and systemic health is an intriguing health issue. PBL components and HC are demonstrated to exhibit hepatoprotective, immunomodulatory, anti-platelet, anti-inflammatory and antioxidative effects [12, 21, 22]. In addition, PBL and HC have potential cancer treatment effects toward various cancers [41, 42]. However, the anticancer effect of PBL is not clear. In this study, PBL extract and one major PBL component - HC (at non-toxic concentrations) prevent the ANE-induced MMP-9 production in oral cancer epithelial cells, suggesting attenuation effects to cancer invasion and metastasis. This is possibly due to antioxidant or other effects, but not through inhibition of COX enzyme activity, because aspirin showed little effect on ANE-induced MMP-9 production in this study. More studies are necessary to further clarify the pharmacological effects of PBL and HC.

Melatonin is secreted from pineal gland and has physiological regulation of sleep and circadian rhythms [43]. It is suggested for use in prevention and treatment of various cancer including head and neck SCC, possibly via effects on angiogenesis, cancer cell proliferation, apoptosis, matrix turnover, matrix metalloproteinase activity and epithelial-mesenchymal transition that are important in primary tumor and cancer metastasis [43, 44]. To further clarify the anticarcinogenic effect of melatonin, we found that melatonin prevents the ANE-induced MMP-9 mRNA expression/secretion of oral cancer cells. Why melatonin inhibits ANE-induced MMP-9 awaits further investigation. The potential mechanisms are blocking of signaling transduction molecules, its antioxidant property and prevent free radicals-mediated cell damage.

## CONCLUSIONS

**Figure 8:**
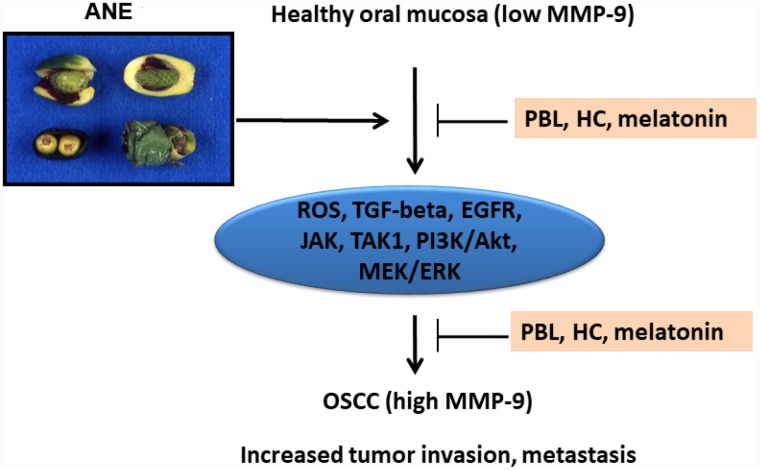
**Proposed signaling mechanisms of ANE-induced MMP-9 expression/ secretion of oral mucosal cells, contribution to oral carcinogenesis and its prevention by PBL, HC and melatonin.**

In conclusion, these results indicate that ANE components (BQ chewing) may enhance tumor invasion and metastasis via stimulation of MMP-9 mRNA expression and secretion. This event is associated with ROS, TGF-β1/Smad-dependent (Smad2), TAK1 and Smad-independent (EGFR, JAK, PI3K/Akt, MEK/ERK) signaling pathways, but not COX activation. ROS scavengers and various small molecules such as PBL, HC, melatonin or antibody for target therapy of these signal transduction pathways can be used for prevention and treatment of BQ chewing-related oral cancer and other diseases (Figure 8).

## MATERIALS AND METHODS

### Materials and chemicals

Dulbecco’s modified Eagles’ medium (DMEM), penicillin/streptomycin, trypsin/EDTA, fetal bovine serum (FBS), KGM-SFM medium, and phosphate-buffered saline (PBS) were from Gibco (Life Technologies, USA). Arecoline, melatonin and MTT were from Sigma (Sigma-Aldrich Chemical Company, St. Louis, MO, USA). ELISA kits for MMP-9 were from PeproTech (PeproTech, Inc., Rocky Hill, NJ, USA). Catalase, SB431542, 5Z-7-Oxozeaenol, PD153035, AG490, U0126, LY294002, and aspirin were purchased from Tocris. Phospho-TAK1 (p-TAK1) antibody was from Abcam (ab79583). Antibodies for TGF-β1, and GAPDH were from Santa Cruz. P-Smad2 antibody was from Cell Signaling Technology. PBL extract and ANE were prepared as before [8, 12, 13, 21]. HC was synthesized as previously [22].

### Culture of GK and SAS cancer epithelial cells

GK were cultured as previously in KGM-SFM with supplements [3, 11], by the approval of Ethics Committee, National Taiwan University Hospital (Ethical Approval Number: 201012090RC, 201112153 RID, 201512152RINC). SAS tongue cancer epithelial cells were cultured in DMEM containing 10% FBS and penicillin/streptomycin. They were regularly subcultured before confluence for use.

### Cytotoxicity of ANE and arecoline to SAS cells

Briefly 5 × 10^5^ cells were inoculated onto 6-well culture wells in 2 ml medium. After 24 hours, medium was changed with fresh medium containing various concentrations of ANE (50-800 μg/ml) or arecoline (0.05–0.8 mM) for 3 days. Medium was collected for ELISA analysis. Cells were washed with PBS and then incubated in medium containing 0.5 mg/ml MTT for 3 hours. Medium was removed and the insoluble formazan generated was dissolved in dimethylsulfoxide (DMSO) and read against blank (DMSO) at OD540 by a Microplate Spectrophotometer.

In some experiments, cells were pretreated to various inhibitors, SB431542 (1 and 5 μM), 5Z-7-Oxozeaenol (1 and 5 μM), catalase (1000 and 2000 U/ml), PD153035 (1 and 5 μM), AG490 (15 and 30 μM), U0126 (10 and 20 μM), LY294002 (10 and 20 μM), PBL extract (500 and 1000 μg/ml), HC (50 and 100 μM), aspirin (100 and 200 μM), melatonin (100 and 250 μg/ml), for 30 min before the addition of ANE. Cells were further co-incubated for 3 days before collection of medium and MTT assay [8, 11].

### Effect of ANE and arecoline on MMP-9 gene expression of SAS Cells: Role of some inhibitors

Briefly 5 x 10^5^ cells were inoculated onto 6-well culture wells in 2 ml medium. After 24 hours, medium was changed with fresh medium containing various concentrations of ANE (100-800 μg/ml) or arecoline (0.05-0.8 mM) for 24 hours. Culture medium was collected for ELISA analysis of MMP-9 secretion as before [8, 45, 46]. Total RNA was isolated for semi-quantitative RT-PCR.

In some experiments, cells were exposed to different concentrations of some inhibitors (PD153035, AG490, 5Z-7-Oxozeaenol, LY294002, PBL, melatonin) for 30 min before the addition of ANE (800 μg/ml). Cells were further co-incubated for 3 days. Medium was collected for ELISA analysis. Cell layers were used for MTT assay for estimation of cytotoxicity. Cell layers were used for RNA isolation.

### Semi-quantitative reverse transcription-polymerase chain reaction

Reverse transcription-polymerase chain reaction (RT-PCR) was done as described before [12]. In short, 3 μg of total RNA was reverse transcribed in a total volume of 44.5 μl reaction solution comprising 8 μl of deoxyribonucleotide triphosphate (dNTP) (2.5 mM), 4 μl of random primer (500 μg/μl), 1 μl of RNase inhibitor (40 U/μl), 4.5 μl of 10x RT buffer, and 0.5 μl of RT (21 U/μl) at 42°C for 90 minutes. Thereafter, 4 μl of complementary deoxyribonucleic acid (cDNA) was taken for PCR amplification in a reaction mixture of 50 μl containing 4 μl of dNTP (2.5 mM), 5 μl of 10x Super Taq buffer, 0.2 μl of Super Taq enzyme (2 U/μl) and 1 μl of each specific primer. The PCR program was set at 94°C for 5 minutes for the first cycle, and then further amplified for 20-35 cycles at 94°C for 1 min, 55°C for 1 min, and then 72°C for 2 min by a thermal cycler (Perkin Elmer 4800, PE Applied Biosystems, Foster City, CA, USA). Finally, the reaction was completed at 72°C for an additional 10 min. The specific primer pairs for β-actin (218 bp) was described previously and used as control [12]. The MMP-9 primer sequence was GTGGTAGCGCACCAGAGGCG and ACCGCCAACTACGA CCGGGA with amplified product of 179 bp. The PCR-generated products were loaded into 1.8% agarose gel electrophoresis with reference of molecular weight markers ranging 100-1200 bp. The gels were finally stained with ethidium bromide and photographed for presentation.

### Effect of inhibitors on ANE-induced MMP-9 production and cytotoxicity of oral epithelial cells

Briefly 5 x 10^5^ cells were inoculated onto 6-well culture wells in 2 ml medium. After 24 hours, medium was changed with fresh medium containing different concentrations of various inhibitors (SB431542, 5Z-7-Oxozeaenol, catalase, PD153035, AG490, LY294002, U0126, PBL extract, HC or aspirin) for 30 min before the addition of ANE (800 μg/ml). Cells were further co-incubated for 3 days. Medium was collected for ELISA analysis [8, 41, 42]. Cell layers were used for MTT assay for estimation of cytotoxicity [8, 11].

### Effect of ANE on TGF-β1, p-Smad2, and p-TAK1 protein expression

### Western blotting analysis

Briefly near confluent GK (in 6-well) or SAS cells (1 × 10^6^ cells) were cultured on 10-cm culture dishes. They were then exposed to different concentrations of ANE (100–800 μg/ml) for 24 hours. Protein from cell lysates were collected and subjected to western blotting analysis as described previously [11, 12], except that TGF-β1 and p-Smad2 antibodies were used as primary antibodies. In some experiments, SB431542 was used for pretreatment and co-incubation with ANE (800 μg/ml) for 24 hours before cell harvesting.

### Immunofluorescent staining analysis

Briefly SAS cells (1 × 10^5^ cells/well) were cultured on coverslips in 24-well culture. They were then exposed to ANE (800 μg/ml) for 5–120 min. Immunofluorescence staining of p-TAK1 expression was performed as before [45]. Medium was decanted, and cells were rinsed by PBS and fixed by paraformaldehyde (4%) for 20 min. Cells were then rinsed by PBS, permeabilized by Triton X-100 (2%), incubated in 0.3% v/v H_2_O_2_ for 20 min. After washed by PBS, bovine serum albumin (BSA, 5%) was applied for blocking of non-specific binding for 1 h, and cells were further incubated at room temperature to primary antibodies (p-TAK1) for overnight. Cells were washed with PBS and then incubated in TRITC-conjugated secondary antibodies (red fluorescence) in the dark for 1 h and counterstained by DAPI (1:1000) of nucleus staining for 30 min. Cells in coverslips were finally mounted and photographed/observed under an inverted microscope and DP Controller/Manager software (Olympus IX71, Olympus Corporation)

### Statistical analysis

At least four or more independent experiments were conducted. If necessary, the results of cell study were analyzed by One-way ANOVA and post-hoc Turkey test.

### Ethics approval

This study is approved by the Ethics Committee, National Taiwan University Hospital.

## References

[1] Jeng JH, Chang MC, Hahn LJ. Role of areca nut in betel quid-associated chemical carcinogenesis: current awareness and future perspectives. Oral Oncol. 2001; 37:477–92. 10.1016/S1368-8375(01)00003-3 11435174

[2] IARC Monograph on the Evaluation of Carcinogenic Risks to Humans. “Betel-quid and Areca-nut chewing and Some Areca Nut-related Nitrosamines” in IARC Monograph. Volume 85. Lyon, France: IARC; 2004 10.1016/0278-6915(86)90083-9 PMC4781453 15635762

[3] Jeng JH, Ho YS, Chan CP, Wang YJ, Hahn LJ, Lei D, Hsu CC, Chang MC. Areca nut extract up-regulates prostaglandin production, cyclooxygenase-2 mRNA and protein expression of human oral keratinocytes. Carcinogenesis. 2000; 21:1365–70. 10.1093/carcin/21.7.1365 10874015

[4] Lin CW, Tseng SW, Yang SF, Ko CP, Lin CH, Wei LH, Chien MH, Hsieh YS. Role of lipocalin 2 and its complex with matrix metalloproteinase-9 in oral cancer. Oral Dis. 2012; 18:734–40. 10.1111/j.1601-0825.2012.01938.x 22533572

[5] Yang JS, Lin CW, Hsieh YH, Chien MH, Chuang CY, Yang SF. Overexpression of carbonic anhydrase IX induces cell motility by activating matrix metalloproteinase-9 in human oral squamous cell carcinoma cells. Oncotarget. 2017; 8:83088–99. 10.18632/oncotarget.20236 29137326 PMC5669952

[6] Tsai CH, Hsieh YS, Yang SF, Chou MY, Chang YC. Matrix metalloproteinase 2 and matrix metalloproteinase 9 expression in human oral squamous cell carcinoma and the effect of protein kinase C inhibitors: preliminary observations. Oral Surg Oral Med Oral Pathol Oral Radiol Endod. 2003; 95:710–16. 10.1067/moe.2003.121 12789153

[7] Tseng YH, Chang KW, Liu CJ, Lin CY, Yang SC, Lin SC. Areca nut extract represses migration and differentiation while activating matrix metalloproteinase -9 of normal gingival epithelial cells. J Periodontal Res. 2008; 43:490–99. 10.1111/j.1600-0765.2007.01035.x 18624942

[8] Chang MC, Chan CP, Wang WT, Chang BE, Lee JJ, Tseng SK, Yeung SY, Hahn LJ, Jeng JH. Toxicity of areca nut ingredients: activation of CHK1/CHK2, induction of cell cycle arrest, and regulation of MMP-9 and TIMPs production in SAS epithelial cells. Head Neck. 2013; 35:1295–302. 10.1002/hed.23119 22907745

[9] Liu SY, Lin MH, Yang SC, Huang GC, Chang L, Chang S, Yen CY, Chiang WF, Lee CH, Kuo YY, Liu YC. Areca quid chewing enhances the expression of salivary matrix metalloproteinase-9. J Formos Med Assoc. 2005; 104:113–19. 15765166

[10] Chang MC, Ho YS, Lee PH, Chan CP, Lee JJ, Hahn LJ, Wang YJ, Jeng JH. Areca nut extract and arecoline induced the cell cycle arrest but not apoptosis of cultured oral KB epithelial cells: association of glutathione, reactive oxygen species and mitochondrial membrane potential. Carcinogenesis. 2001; 22:1527–35. 10.1093/carcin/22.9.1527 11532876

[11] Chang MC, Wu HL, Lee JJ, Lee PH, Chang HH, Hahn LJ, Lin BR, Chen YJ, Jeng JH. The induction of prostaglandin E2 production, interleukin-6 production, cell cycle arrest, and cytotoxicity in primary oral keratinocytes and KB cancer cells by areca nut ingredients is differentially regulated by MEK/ERK activation. J Biol Chem. 2004; 279:50676–83. 10.1074/jbc.M404465200 15375172

[12] Chang MC, Chen YJ, Chang HH, Chan CP, Yeh CY, Wang YL, Cheng RH, Hahn LJ, Jeng JH. Areca nut components affect COX-2, cyclin B1/cdc25C and keratin expression, PGE2 production in keratinocyte is related to reactive oxygen species, CYP1A1, Src, EGFR and Ras signaling. PLoS One. 2014; 9:e101959. 10.1371/journal.pone.0101959 25051199 PMC4106785

[13] Chang MC, Chan CP, Chen YJ, Hsien HC, Chang YC, Yeung SY, Jeng PY, Cheng RH, Hahn LJ, Jeng JH. Areca nut components stimulate ADAM17, IL-1α, PGE2 and 8-isoprostane production in oral keratinocyte: role of reactive oxygen species, EGF and JAK signaling. Oncotarget. 2016; 7:16879–94. 10.18632/oncotarget.7621 26919242 PMC4941357

[14] Tseng YH, Chang CS, Liu TY, Kao SY, Chang KW, Lin SC. Areca nut extract treatment down-regulates involucrin in normal human oral keratinocyte through P13K/AKT activation. Oral Oncol. 2007; 43:670–79. 10.1016/j.oraloncology.2006.08.003 17070098

[15] Moutasim KA, Jenei V, Sapienza K, Marsh D, Weinreb PH, Violette SM, Lewis MP, Marshall JF, Fortune F, Tilakaratne WM, Hart IR, Thomas GJ. Betel-derived alkaloid up-regulates keratinocyte alphavbeta6 integrin expression and promotes oral submucous fibrosis. J Pathol. 2011; 223:366–77. 10.1002/path.2786 21171082

[16] Pant I, Rao SG, Kondaiah P. Role of areca nut induced JNK/ATF2/Jun axis in the activation of TGF-β pathway in precancerous Oral Submucous Fibrosis. Sci Rep. 2016; 6:34314. 10.1038/srep34314 27708346 PMC5052620

[17] Onodera Y, Teramura T, Takehara T, Shigi K, Fukuda K. Reactive oxygen species induce Cox-2 expression via TAK1 activation in synovial fibroblast cells. FEBS Open Bio. 2015; 5:492–501. 10.1016/j.fob.2015.06.001 26110105 PMC4476901

[18] Fechtner S, Fox DA, Ahmed S. Transforming growth factor β activated kinase 1: a potential therapeutic target for rheumatic diseases. Rheumatology (Oxford). 2017; 56:1060–68. 10.1093/rheumatology/kew301 27550296 PMC5850516

[19] Nishimura M, Shin MS, Singhirunnusorn P, Suzuki S, Kawanishi M, Koizumi K, Saiki I, Sakurai H. TAK1-mediated serine/threonine phosphorylation of epidermal growth factor receptor via p38/extracellular signal-regulated kinase: NF-{kappa}B-independent survival pathways in tumor necrosis factor alpha signaling. Mol Cell Biol. 2009; 29:5529–39. 10.1128/MCB.00375-09 19687304 PMC2756876

[20] Mihaly SR, Ninomiya-Tsuji J, Morioka S. TAK1 control of cell death. Cell Death Differ. 2014; 21:1667–76. 10.1038/cdd.2014.123 25146924 PMC4211365

[21] Jeng JH, Chen SY, Liao CH, Tung YY, Lin BR, Hahn LJ, Chang MC. Modulation of platelet aggregation by areca nut and betel leaf ingredients: roles of reactive oxygen species and cyclooxygenase. Free Radic Biol Med. 2002; 32:860–71. 10.1016/S0891-5849(02)00749-9 11978487

[22] Chang MC, Uang BJ, Tsai CY, Wu HL, Lin BR, Lee CS, Chen YJ, Chang CH, Tsai YL, Kao CJ, Jeng JH. Hydroxychavicol, a novel betel leaf component, inhibits platelet aggregation by suppression of cyclooxygenase, thromboxane production and calcium mobilization. Br J Pharmacol. 2007; 152:73–82. 10.1038/sj.bjp.0707367 17641677 PMC1978281

[23] Reiter RJ, Rosales-Corral SA, Tan DX, Acuna-Castroviejo D, Qin L, Yang SF, Xu K. Melatonin, a full service anti-cancer agent: inhibition of initiation, progression and metastasis. Int J Mol Sci. 2017; 18:E843. 10.3390/ijms18040843 28420185 PMC5412427

[24] Su SC, Reiter RJ, Hsiao HY, Chung WH, Yang SF. Functional interaction between melatonin signaling and nocoding RNAs. Trends Endocrinol Metab. 2018; 29:435–45. 10.1016/j.tem.2018.03.008 29631868

[25] Adel M, Liao CT, Lee LY, Hsueh C, Lin CY, Fan KH, Wang HM, Ng SH, Lin CH, Tsao CK, Huang SF, Kang CJ, Fang KH, et al. Incidence and outcomes of patients with oral cavity squamous cell carcinoma and fourth primary tumors: A long-term follow-up study in a betel quid chewing endemic area. Medicine (Baltimore). 2016; 95:e2950. 10.1097/MD.0000000000002950 27015170 PMC4998365

[26] Smith PC, Cáceres M, Martínez C, Oyarzún A, Martínez J. Gingival wound healing: an essential response disturbed by aging? J Dent Res. 2015; 94:395–402. 10.1177/0022034514563750 25527254 PMC4814024

[27] Daniele A, Abbate I, Oakley C, Casamassima P, Savino E, Casamassima A, Sciortino G, Fazio V, Gadaleta-Caldarola G, Catino A, Giotta F, De Luca R, Divella R. Clinical and prognostic role of matrix metalloproteinase-2, -9 and their inhibitors in breast cancer and liver diseases: A review. Int J Biochem Cell Biol. 2016; 77:91–101. 10.1016/j.biocel.2016.06.002 27267661

[28] Gonzalez-Avila G, Sommer B, Mendoza-Posada DA, Ramos C, Garcia-Hernandez AA, Falfan-Valencia R. Matrix metalloproteinases participation in the metastatic process and their diagnostic and therapeutic applications in cancer. Crit Rev Oncol Hematol. 2019; 137:57–83. 10.1016/j.critrevonc.2019.02.010 31014516

[29] Reichart PA, Phillipsen HP. Betel chewer’s mucosa—a review. J Oral Pathol Med. 1998; 27:239–42. 10.1111/j.1600-0714.1998.tb01949.x 9707274

[30] Yang Y, Qiu Y, Tang M, Wu Z, Hu W, Chen C. Expression and function of transforming growth factor-β-activated protein kinase 1 in gastric cancer. Mol Med Rep. 2017; 16:3103–10. 10.3892/mmr.2017.6998 28714004 PMC5548047

[31] Huang HL, Chiang CH, Hung WC, Hou MF. Targeting of TGF-β-activated protein kinase 1 inhibits chemokine (C-C motif) receptor 7 expression, tumor growth and metastasis in breast cancer. Oncotarget. 2015; 6:995–1007. 10.18632/oncotarget.2739 25557171 PMC4359270

[32] Xing S, Yu W, Zhang X, Luo Y, Lei Z, Huang D, Lin J, Huang Y, Huang S, Nong F, Zhou C, Wei G. Isoviolanthin extracted from Dendrobium officinale reverses TGF-β1-mediated epithelial-mesenchymal transition in hepatocellular carcinoma cells via deactivating the TGF-/Smad and PI3K/Akt/mTOR signaling pathways. Int J Mol Sci. 2018; 19:E1556. 10.3390/ijms19061556 29882900 PMC6032198

[33] Tyszka-Czochara M, Lasota M, Majka M. Caffeic acid and metformin inhibit invasive phenotype induced by TGF-β1 in C-4I and HTB-35/SiHa cervical squamous carcinoma cells by acting on different molecular targets. Int J Mol Sci. 2018; 19:E266. 10.3390/ijms19010266 29337896 PMC5796212

[34] Wang B, Wang XB, Chen LY, Huang L, Dong RZ. Belinostat-induced apoptosis and growth inhibition in pancreatic cancer cells involve activation of TAK1-AMPK signaling axis. Biochem Biophys Res Commun. 2013; 437:1–6. 10.1016/j.bbrc.2013.05.090 23743198

[35] Liu RY, Zeng Y, Lei Z, Wang L, Yang H, Liu Z, Zhao J, Zhang HT. JAK/STAT3 signaling is required for TGF-β-induced epithelial-mesenchymal transition in lung cancer cells. Int J Oncol. 2014; 44:1643–51. 10.3892/ijo.2014.2310 24573038

[36] Hashmi AA, Hussain ZF, Aijaz S, Irfan M, Khan EY, Naz S, Faridi N, Khan A, Edhi MM. Immunohistochemical expression of epidermal growth factor receptor (EGFR) in South Asian head and neck squamous cell carcinoma: association with various risk factors and clinico-pathologic and prognostic parameters. World J Surg Oncol. 2018; 16:118. 10.1186/s12957-018-1425-3 29954411 PMC6022491

[37] Suwanwela J, Osathanon T. Inflammation related genes are upregulated in surgical margins of advanced stage oral squamous cell carcinoma. J Oral Biol Craniofac Res. 2017; 7:193–97. 10.1016/j.jobcr.2017.05.003 29123999 PMC5670299

[38] Wu HT, Ko SY, Fong JH, Chang KW, Liu TY, Kao SY. Expression of phosphorylated Akt in oral carcinogenesis and its induction by nicotine and alkaline stimulation. J Oral Pathol Med. 2009; 38:206–13. 10.1111/j.1600-0714.2008.00659.x 18331557

[39] Yang SF, Tsai CH, Chang YC. The upregulation of heat shock protein 47 expression in human buccal fibroblasts stimulated with arecoline. J Oral Pathol Med. 2008; 37:206–10. 10.1111/j.1600-0714.2007.00633.x 18221324

[40] Lee SS, Tseng LH, Li YC, Tsai CH, Chang YC. Heat shock protein 47 expression in oral squamous cell carcinomas and upregulated by arecoline in human oral epithelial cells. J Oral Pathol Med. 2011; 40:390–96. 10.1111/j.1600-0714.2010.00998.x 21198874

[41] Jeng JH, Wang YJ, Chang WH, Wu HL, Li CH, Uang BJ, Kang JJ, Lee JJ, Hahn LJ, Lin BR, Chang MC. Reactive oxygen species are crucial for hydroxychavicol toxicity toward KB epithelial cells. Cell Mol Life Sci. 2004; 61:83–96. 10.1007/s00018-003-3272-8 14704856 PMC11138776

[42] Gundala SR, Yang C, Mukkavilli R, Paranjpe R, Brahmbhatt M, Pannu V, Cheng A, Reid MD, Aneja R. Hydroxychavicol, a betel leaf component, inhibits prostate cancer through ROS-driven DNA damage and apoptosis. Toxicol Appl Pharmacol. 2014; 280:86–96. 10.1016/j.taap.2014.07.012 25064160 PMC4363134

[43] Yeh CM, Su SC, Lin CW, Yang WE, Chien MH, Reiter RJ, Yang SF. Melatonin as a potential inhibitory agent in head and neck cancer. Oncotarget. 2017; 8:90545–56. 10.18632/oncotarget.20079 29163852 PMC5685773

[44] Su SC, Hsieh MJ, Yang WE, Chung WH, Reiter RJ, Yang SF. Cancer metastasis: Mechanisms of inhibition by melatonin. J Pineal Res. 2017; 62. 10.1111/jpi.12370 27706852

[45] Chang MC, Chen YJ, Lian YC, Chang BE, Huang CC, Huang WL, Pan YH, Jeng JH. Butyrate stimulates histone H3 acetylation, 8-isoprostane production, RANKL expression, and regulated osteoprotegerin expression/secretion in MG-63 osteoblastic cells. Int J Mol Sci. 2018; 19:E4071. 10.3390/ijms19124071 30562925 PMC6321057

[46] Chang MC, Chang BE, Pan YH, Lin BR, Lian YC, Lee MS, Yeung SY, Lin LD, Jeng JH. Antiplatelet, antioxidative, and anti-inflammatory effects of hydroquinone. J Cell Physiol. 2019; 234:18123–30. 10.1002/jcp.28444 30843219

